# Association between risk of preterm birth and long-term and short-term exposure to ambient carbon monoxide during pregnancy in chongqing, China: a study from 2016-2020

**DOI:** 10.1186/s12889-024-18913-z

**Published:** 2024-05-27

**Authors:** Xin Ming, Yunping Yang, Yannan Li, Ziyi He, Xiaoqin Tian, Jin Cheng, Wenzheng Zhou

**Affiliations:** 1 Department of Quality Management Section, Women and Children’s Hospital of Chongqing Medical University, Chongqing, 401147 China; 2 Department of Quality Management Section, Chongqing Health Center for Women and Children, Chongqing, 401147 China; 3 Chongqing Research Center for Prevention & Control of Maternal and Child Disease and Public Health, Chongqing, China; 4 Department of Epidemiology, School of Public Health, Chongqing Medical University, Chongqing, China; 5 Department of Public Health and Emergency Management, Chongqing Medical and Pharmaceutical College, Chongqing, China

**Keywords:** Air pollution, CO, Preterm birth, Very preterm birth

## Abstract

**Background:**

Preterm birth (PTB) is an important predictor of perinatal morbidity and mortality. Previous researches have reported a correlation between air pollution and an increased risk of preterm birth. However, the specific relationship between short-term and long-term exposure to carbon monoxide (CO) and preterm birth remains less explored.

**Methods:**

A population-based study was conducted among 515,498 pregnant women in Chongqing, China, to assess short-term and long-term effects of CO on preterm and very preterm births. Generalized additive models (GAM) were applied to evaluate short-term effects, and exposure-response correlation curves were plotted after adjusting for confounding factors. Hazard ratios (HR) and 95% confidence intervals (CI) were calculated using COX proportional hazard models to estimate the long-term effect.

**Results:**

The daily incidence of preterm and very preterm birth was 5.99% and 0.41%, respectively. A positive association between a 100 µg/m³ increase in CO and PTB was observed at lag 0–3 days and 12–21 days, with a maximum relative risk (RR) of 1.021(95%CI: 1.001–1.043). The exposure-response curves (lag 0 day) revealed a rapid increase in PTB due to CO. Regarding long-term exposure, positive associations were found between a 100 µg/m^3^ CO increase for each trimester(Model 2 for trimester 1: HR = 1.054, 95%CI: 1.048–1.060; Model 2 for trimester 2: HR = 1.066, 95%CI: 1.060–1.073; Model 2 for trimester 3: HR = 1.007, 95%CI: 1.001–1.013; Model 2 for entire pregnancy: HR = 1.080, 95%CI: 1.073–1.088) and higher HRs of very preterm birth. Multiplicative interactions between air pollution and CO on the risk of preterm and very preterm birth were detected (*P*- interaction<0.05).

**Conclusions:**

Our findings suggest that short-term exposure to low levels of CO may have protective effects against preterm birth, while long-term exposure to low concentrations of CO may reduce the risk of both preterm and very preterm birth. Moreover, our study indicated that very preterm birth is more susceptible to the influence of long-term exposure to CO during pregnancy, with acute CO exposure exhibiting a greater impact on preterm birth. It is imperative for pregnant women to minimize exposure to ambient air pollutants.

**Supplementary Information:**

The online version contains supplementary material available at 10.1186/s12889-024-18913-z.

## Introduction

According to the definition provided by the World Health Organization (WHO), preterm birth refers to the delivery of a live baby before completing 37 weeks of gestation, with subcategories including very preterm birth (VPTB, < 32 weeks gestation) [1]. Numerous studies have demonstrated that preterm birth (PTB) serves as a crucial predictor of perinatal morbidity and mortality [2, 3]. Infants born before 25 weeks of gestation face a 40% likelihood of mortality before discharge [4]. By 34 weeks, only 50% of preterm infants exhibit a survival rate, and at the age of 2 years old, approximately 4% manifest cerebral palsy, while 8% experience neurological delays [4–6]. Furthermore, preterm birth affects 5 to 11% of newborns globally, varying by ethnicity, country, or other factors [7].

Air pollution has emerged as a significant environmental hazard to human health. The 2017 Global Burden of Diseases (GBD) Study revealed that over 4.9 million preterm births worldwide could be attributable to ambient air pollution, with 1.2 million occurring in China alone [8]. Our prior investigations delved into the correlation between exposure to ambient air pollutants and adverse birth outcomes [9–12]. The exposure assessment relied on measurements obtained from 17 ground monitoring stations located in Chongqing. Our research found that elevated levels of PM_2.5_ and PM_10_ were linked to higher risk of spontaneous abortion for short-term effects [12]. Additionally, we reported that maternal exposure to high levels of PM_2.5_ and PM_10_ during pregnancy may increase risk for preterm birth at late stage of pregnancy. There was important to note that exposure to CO significantly increased the risk of preterm birth during each trimester and entire pregnancy [11].

CO, a colorless, odorless gas resulting from the incomplete combustion of fossil fuels, has been the focus of epidemiological literature linking environmental fine particulate matter to an elevated risk of preterm birth [13–16]. A prospective birth cohort study found 15% increases in risk of PTB with 100 µg/m^3^ increase in CO mean concentrations [17]. However, a research from Southern California suggests that increased concentrations of CO in the environment may (to a lesser extent) contribute to the occurrence of preterm birth [18].

Few researchers have specifically explored the relationship between CO and preterm birth, particularly very preterm birth. The present study concentrates specifically on evaluating the impact of carbon monoxide (CO) on preterm birth. This investigation not only expands upon the earlier analysis of preterm birth but also introduces results about very preterm birth and maternal age stratification to calculate the hazard risk (HR). Consequently, our focus in this research is to explore and assess both short-term and long-term effects of ambient CO on preterm birth.

## Materials and methods

### Study area

Chongqing is a large municipality located in southwest China, under the direct control of the national government. It is situated between longitudes 28°10′ N-32°13′ N and latitudes 105°11′ E -110°11′ E. It covers an area of 82,400 square kilometers. Moreover, Chongqing is nicknamed “Fog City” due to its basin topography and meteorological conditions that hinder the diffusion of air pollutants. According to the seventh population census of China, the resident population of Chongqing is 32.12 million. The research area of this paper was the main urban area of Chongqing, including nine closely connected districts: Yubei, Jiulongpo, Jiangbei, Yuzhong, Dadukou, Shapingba, Nanan, Banan and Beibei.

### Study population

This is a retrospective cohort study using 5-year daily data. The birth outcome data was obtained from the Chongqing Birth Certificate System, including the gestational age, date of birth, maternal address, etc. After excluding those who lacked gestational age and non-urban residents, we obtained 515,498 pregnant women living in the main urban area of Chongqing from 2016 to 2020. After delivery, the baby’s birth information was filled in the neonatal care record system by the health care worker or midwife. The information was then verified and uploaded to the system. Parents and healthcare boards confirm this information before issuing a birth certificate.

### Exposure assessment and outcomes

During the research period, daily 24-hour monitor data of PM_2.5_, PM_10_, SO_2_, NO_2_, CO and O_3_ (8 h maximum value) were collected from 17 ground-based monitoring stations in Chongqing via the Chinese National Urban Air Quality Monitoring Platform (https://zhb.gov.cn). Daily average relative humidity and temperature were available from the China Greenhouse Data Sharing Platform (http://data.sheshiyuanyi.com, accessed on 2 April 2021).

For short-term effect of pollutants, we took the number of preterm and very preterm births per day as the independent variable, and the average daily concentrations of pollutants and meteorological factors as the covariate from the perspective of population.

For long-term exposure to pollutants, each pregnant woman’s exposure to air pollutants and meteorological factors was calculated for each day from fertilization to delivery. Specifically, maternal addresses were geocoded to x and y coordinates with Python through Amap key. Then, we used inverse distance weighting (IDW) to assess each pregnancy woman’s exposure to six pollutants, temperature and relative humidity by ArcGIS during each day of pregnancy. Finally, we averaged the pollutants and meteorological factors of each woman in each trimester and the entire pregnancy period.

### Outcomes

Gestational age at the time of delivery was calculated in weeks from the first day of the last menstrual period. Based on gestational age, preterm birth was defined as a live birth that occurs before 37 completed weeks of gestation [19]. Very preterm birth was defined as a delivery prior to 32 completed weeks of gestation. Full term birth was defined as a birth occurs at 37 or more weeks of gestation.

### Data analysis

Previous researches have shown that the relationship between preterm birth and very preterm birth are small probability events, and have a non-linear relationship with air pollution.

Thus, for short-term effects, we used distributed lag nonlinear model (DLNM) with quasi-Poisson GAM to estimate effect of CO on preterm birth. The equation was as follows:

$${Y_t} \sim \,quasi\, - \,Poission\,{\rm{(}}E{\rm{(}}{Y_t}{\rm{))}}$$

$$\eqalign{ F\, = \, & Log\left( {E({Y_t}} \right)) = \alpha + cb\left( {C{O_t},lag} \right) + cb\left( {Tem{p_t},lag} \right) + \cr & cb\left( {R{H_t},lag} \right) + ns\left( {Tim{e_t},df} \right) + \cr & as.factor\left( {DO{W_t}} \right) + as.factor{\rm{(holida}}{{\rm{y}}_{\rm{t}}}{\rm{)}} \cr}$$

$$\eqalign{{F_{adjust}}\, = \, & Log\left( {E({Y_t}} \right)) = \alpha + cb\left( {C{O_t},lag} \right) + \cr & cb\left( {Tem{p_t},lag} \right) + cb\left( {R{H_t},lag} \right) + \cr & ns\left( {C{O_t},Pollutio{n_t}} \right) + ns\left( {Tim{e_t},df} \right) + \cr & as.factor\left( {DO{W_t}} \right) + as.factor{\rm{(holida}}{{\rm{y}}_{\rm{t}}}{\rm{)}} \cr}$$

Where ${Y}_{t}$ is the observed daily preterm birth counts at day t; $\propto$ is the model intercept; ${Pollution}_{t}$ stands for the pollutant concentration at day $t$, ${Temp}_{t}$ represents the temperature on day $t$, ${RH}_{t}$ represents the relative humidity on day $t$; $cb\left({Temp}_{t},lag\right)$, $cb\left({RH}_{t},lag\right)$ indicates the matrix of temperature and relative humidity. $s\left({CO}_{t},{Pollution}_{t}\right)$ is the interaction effect of CO and air pollution. Then we use the DLNMs by the definition of a “cross-basis” function, a two dimensional function space expressing the influence of the predictor range and in its lag dimension. ${Time}_{t}$ is time trend, and *ns()* denotes a natural cubic smooth spline function that eliminates unobserved long-term and seasonal patterns from the dataset of time series. ${DOW}_{t}$ was the day of week; ${holiday}_{t}$ represent dummy variable (0 indicates non-holiday, and 1 indicates a holiday).

For the long-term effects, we used Cox proportional hazards models to assess the hazard ratios (HRs) of preterm birth with CO exposure in each trimester and entire pregnancy period, respectively. The time scale used gestational age which allows for better control of association of preterm birth risk and gestational age. More importantly, the Cox model provides a more straightforward interpretation that translates HRs into “risk” compared to the ratio of the odds from logistic regression. We fitted three models for each exposure time window: an unadjusted model (Model 1); a model adjusted for temperature and relative humidity (Model 2); a model adjusted for interaction term (exposure of CO and other each air pollutant), temperature and relative humidity (Model 3). In model 3, we used a multiplicative interaction term to explore the interactions between CO and other air pollutants (PM_2.5_, PM_10_, NO_2_, SO_2_, O_3_), and *p* < 0.05 of the product term indicates a multiplicative interaction.

To assess potential effect modification by maternal age, we performed analysis stratified by age (≤ 21 years, 22–34 years, and ≥ 35 years), and obtained HRs and 95% confidence intervals (CIs). Furthermore, we employed a restricted cubic spline (RCS) with four knots (ascertained by using the Akaike information criterion) to construct exposure-response curves for the association between long-term CO exposure and risk of incident preterm and very preterm birth. The nonlinearity was assessed using ANOVA F statistic, while the findings were visually presented through a line plot.

In addition to the main analyses, we performed several sensitivity analyses to confirm the robustness of our estimates by: (1) excluding the mothers whose residential address was more than 10 km away from the nearest monitoring station; (2)plotting the exposure-response curves of CO for incident preterm and very preterm birth risk; (3)further adjusting temperature, relative humidity and multiplicative interaction. (4)building a multi-trimester model including CO exposure in first, second and third trimester to check the robustness of the estimated associations. R software (version 4.1.3, R Foundation for Statistical Computing, Vienna, Austria) was used for all analyses, with “mgcv”, “spline” and“dlnm” packages. A probability value of<0.05 was considered statistically significant for all statistical tests.

## Results

### Descriptive statistics of the research objects

We summarized the demographic characteristics of live births in urban of Chongqing, China (Table 1). From January 1 st, 2016 to December 31 st, 2020, a total of 515.498 live births were identified from the birth register system. Among these, 30,884 live births were preterm (5.99%), 2120 were very preterm births (0.41%), and 484,614 were full-term births (94.01%). The incidence of both preterm birth and very preterm birth increases with advanced maternal age, particularly when the mother was 35 years old or older. Notably, both preterm and very preterm births exhibited higher average concentrations of CO during each trimester and throughout the entire pregnancy compared to full-term births. Throughout the entire pregnancy, very preterm births had the highest average level of CO compared to full-term and preterm births.

Supplementary Fig. 1 illustrates the time-series trends of ambient air pollution, daily preterm births and very preterm births from 2013 to 2019. The concentrations of PM_2.5_, PM_10_, SO_2_, NO_2_ and CO exhibited seasonal variations, with higher levels observed during winter months and lower levels during summer. The daily incidence of preterm birth was 16.923, while that of very preterm birth was 1.162. Over the five-year period, the average concentration of PM_2.5_, PM_10_, SO_2_, NO_2_, O_3_, and CO was 40.735 µg/m^3^, 64.003 µg/m^3^, 9.376 µg/m^3^, 40.249 µg/m^3^, 42.368 µg/m^3^ and 886.521 µg/m^3^, respectively. Additionally, the average daily relative humidity was 75.105%, and the mean temperature was 19.972℃ (Supplementary Table 1).

**Table 1:** Select demographic characteristics of live birth data in Chongqing, China from 2016 to 2020

Characteristics	Total births (*n* = 515,498)	Preterm births (*n* = 30,884)	Very preterm births (*n* = 2120)	Full term births (*n* = 484,614)
Gestational age (year)	38.71 ± 1.50	34.73 ± 1.83	29.54 ± 1.60	38.97 ± 1.05
Birth weight (g)	3274.70 ± 461.06	2453.11 ± 531.70	1431.38 ± 382.49	3327.06 ± 402.92
Maternal age	28.88 ± 4.98	30.01 ± 5.08	30.18 ± 5.12	28.81 ± 4.96
≤ 21 years	26,529	1277(4.81%)	92(0.35%)	25,252(95.19%)
22–35 years	421,405	23,843(5.66%)	1600(0.38%)	397,562(94.34%)
≥ 35 years	67,069	5731(8.54%)	428(0.64%)	61,338(91.46%)
Missing	495	33(6.67%)	0(0.00%)	462(93.33%)
**Season of conception**				
Spring	121,628	7296(6.00%)	501(0.41%)	114,332(94.00%)
Summer	121,896	7485(6.14%)	512(0.42%)	114,411(93.86%)
Autumn	141,034	7825(5.54%)	573(0.41%)	133,209(94.45%)
Winter	130,940	8278(6.32%)	534(0.41%)	122,662(93.68%)
**Year**				
2016	99,407	4962(4.99%)	249(0.25%)	94,445(95.01%)
2017	132,027	7449(5.64%)	534(0.40%)	124,578(94.36%)
2018	99,072	6414(6.47%)	424(0.43%)	92,658(93.53%)
2019	104,667	6626(6.33%)	487(0.47%)	98,041(93.67%)
2020	80,325	5433(6.76%)	426(0.53%)	74,892(93.24%)
**CO (µg/m** ^**3**^ **)**				
CO Trimester1 (µg/m^3^)	996.29 ± 229.11	1009.99 ± 230.19	1038.33 ± 229.87	995.42 ± 229.01
CO Trimester2 (µg/m^3^)	1009.41 ± 230.87	1026.52 ± 222.84	1050.66 ± 210.54	1008.32 ± 231.33
CO Trimester3 (µg/m^3^)	1035.86 ± 237.67	1046.22 ± 243.39	1052.00 ± 253.45	1035.20 ± 237.29
CO Entire pregnancy (µg/m^3^)	1010.84 ± 175.86	1024.31 ± 178.15	1048.91 ± 178.82	1009.99 ± 175.68

### Short-term effect

**Fig. 1:**
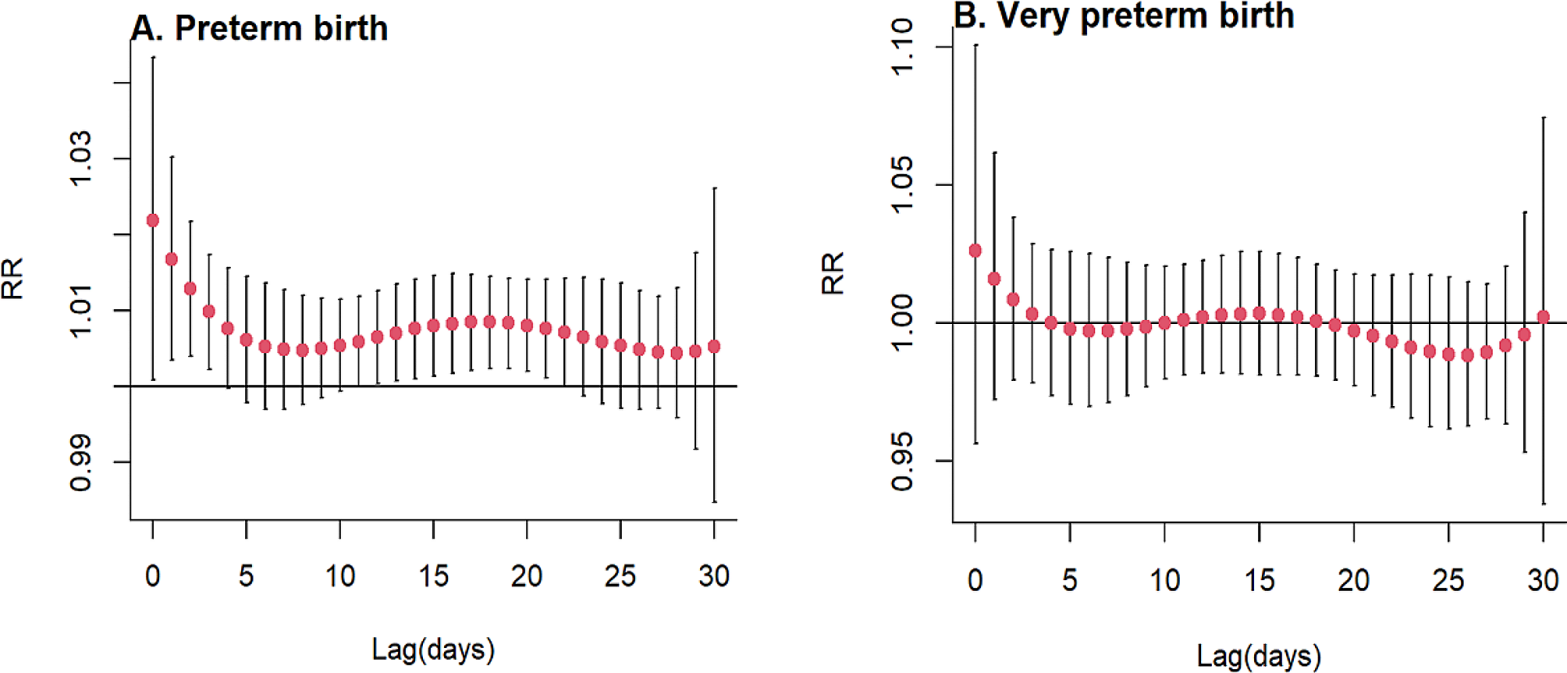
The slice’s plots of the relative risk (95% CI) value of CO with 30 day lag for preterm and very preterm birth

**Fig. 2:**
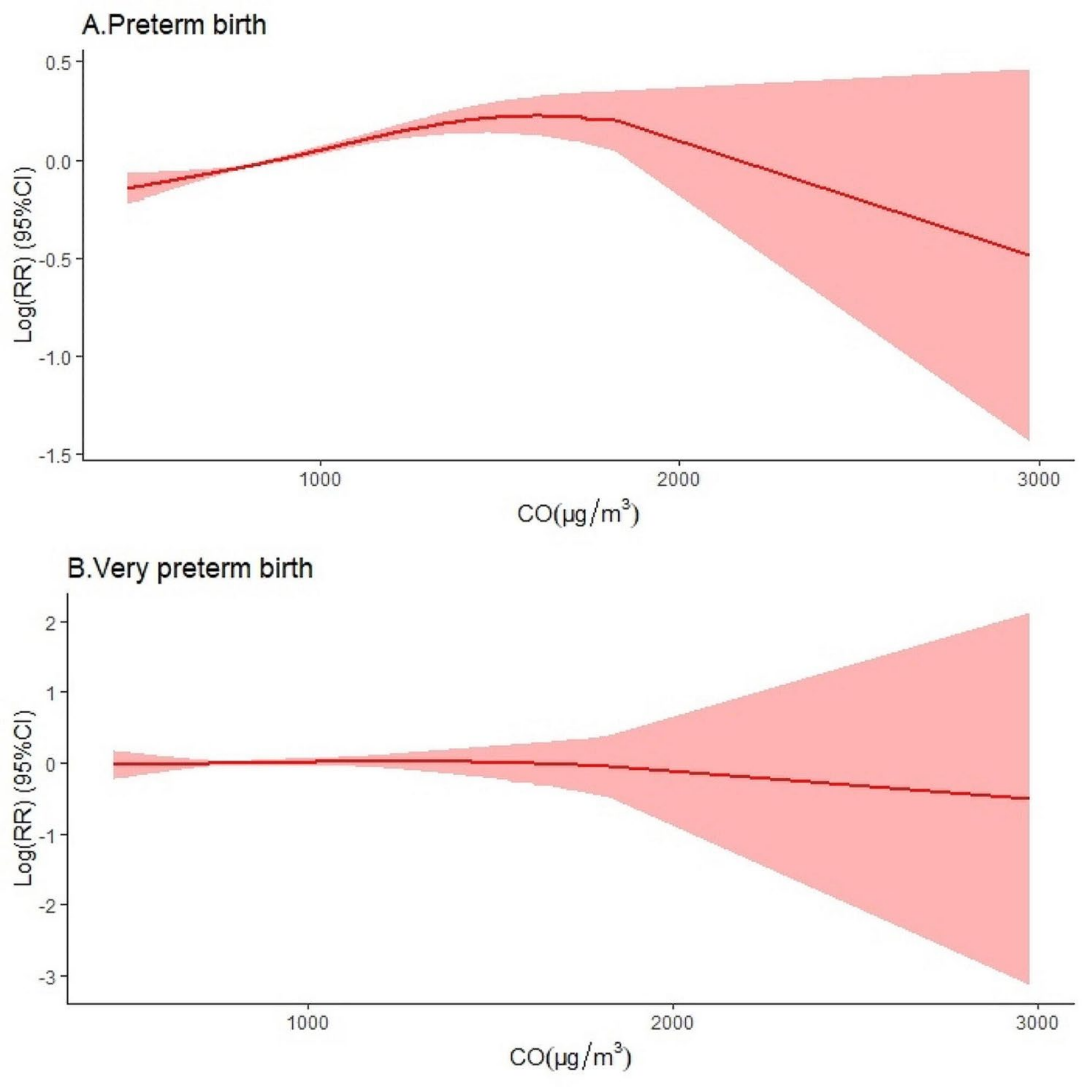
Exposure-response association curves between CO concentration (µg/m^3^, lag0) and risk of preterm birth and very preterm birth. The vertical scale represents the log of relative risk of daily preterm birth and very preterm birth related to a 100 µg/m^3^ increment of ambient CO

We conducted a risk assessment to evaluate the potential acute impact of CO on the rates of preterm and very preterm births, as depicted in Fig. 1. Our findings indicate that pregnant mothers exposed to CO may have an increased likelihood of giving birth prematurely, particularly when exposed during lag 0–3 days and 12–21 days. The maximum RR was 1.021, with a 95% confidence interval ranging from 1.001 to 1.043. However, no significant association with CO was observed for very preterm births. To further elucidate the relationship between the concentrations of CO and preterm and very preterm birth, exposure-response correlation curves were plotted at lag 0 day, as shown in Fig. 2. Notably, the response curve for CO exposure exhibited a protective effect on preterm birth at relatively low levels. Conversely, at higher concentrations, CO posed a risk factor for preterm birth, with a positive correlation between concentration and log(RR) value. No evidence was found for a relationship between CO concentrations and very preterm births on the current day.

### Long-term effect

Tables 2 and 3 present the unadjusted and adjusted hazard risk of preterm and very preterm birth linked to CO exposure during each trimester and the entire pregnancy. Model 2, accounting for temperature and humidity, revealed that CO exposure during the first trimester, second trimester, and entire pregnancy had a greater impact on preterm birth compared to Model 1. Each 100 µg/m^3^ increase in CO concentration before conception was significantly associated with preterm birth (Model 2 for trimester 1: HR = 1.054, 95%CI: 1.048–1.060; Model 2 for trimester 2: HR = 1.066, 95%CI: 1.060–1.073; Model 2 for trimester 3: HR = 1.007, 95%CI: 1.001–1.013; Model 2 for entire pregnancy: HR = 1.080, 95%CI: 1.073–1.088). The strongest effect for preterm birth was observed in the second trimester for CO, while for very preterm birth it was in the first trimester.

Significantly multiplicative interactions were identified between air pollutants (PM_2.5_, PM_10_, NO_2_ and SO_2_) and CO on the risk of preterm birth and very preterm birth (Tables 2 and 3). The largest HR (95% CI) for preterm birth was associated with the combination of CO and PM_2.5_ in trimester 2 (HR = 1.162, 95%CI: 1.150–1.174). In addition, the combined effect of O_3_ and CO on preterm birth was diminished due to their multiplicative interactions. The adjusted HR (Model 2) for preterm birth with every 100 µg/m3 increase in CO was 1.054 (95%CI: 1.048, 1.060), and the HR value for the interaction between CO and O_3_ during early pregnancy was 1.151 (95%CI: 1.045–1.157). Similar significantly multiplicative interactions were found between individual air pollutants and CO on the risk of very preterm birth. Moreover, the effect of CO on very preterm birth was higher than that on preterm birth at the same time and model. Interestingly, the impact of CO in the third trimester, whether on preterm or very preterm birth, was found to be minimal.

**Fig. 3:**
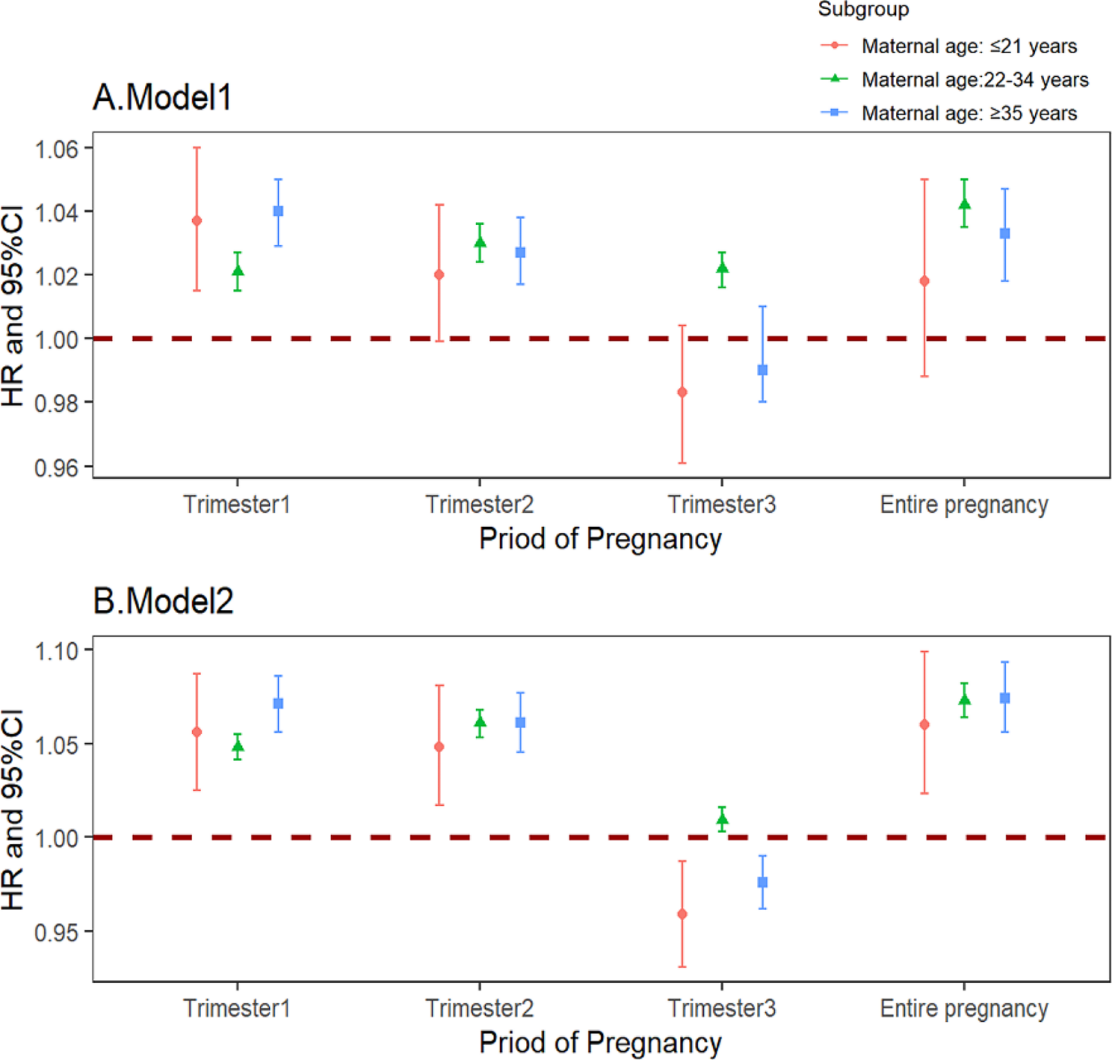
The HRs of long-term exposure to CO with the risk of preterm birth in different age groups *Note* Model1 was unadjusted. Model2 was adjusted for temperature, relative humidity

**Fig. 4:**
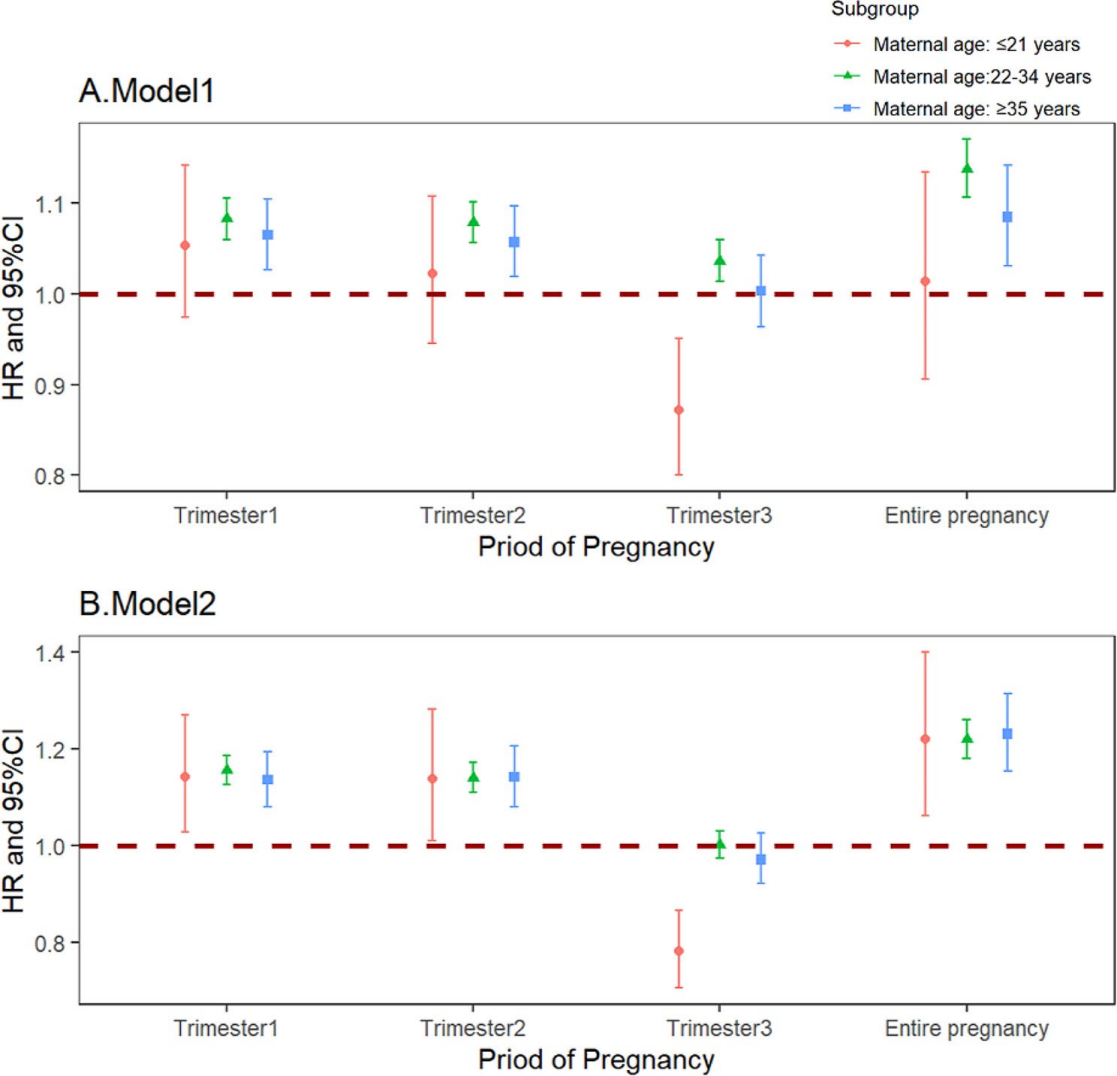
The HRs of long-term exposure to CO with the risk of very preterm birth in different age groups *Note* Model 1 was unadjusted. Model 2 was adjusted for temperature and relative humidity

In the stratified analyses, the risk of preterm and very preterm birth associated with long-term CO exposure varied by maternal age ( Figs. 3 and 4). In model 2, mothers aged over 35 exhibited the highest risk of preterm birth when exposed to CO compared to those aged ≤ 21 and 22–34 in the 1st trimester (HR = 1.071, 95%CI: 1.056–1.086), 2nd trimester (HR = 1.061, 95%CI: 1.045–1.077) and entire pregnancy (HR = 1.074, 95%CI: 1.056–1.093). An intriguing result in the 3rd trimester indicated a protective effect of CO against preterm birth in mothers aged ≤ 21 (HR = 0.959, 95%CI: 0.931–0.987) and ≥ 35 (HR = 0.976, 95%CI: 0.962–0.990). Notably, in the group of mothers aged ≤ 21 (HR = 0.782, 95%CI: 0.706–0.866), CO exposure in the 3rd trimester exhibited a protective effect against very preterm birth, suggesting a potential risk reduction for pregnant women in this age group.

**Fig. 5:**
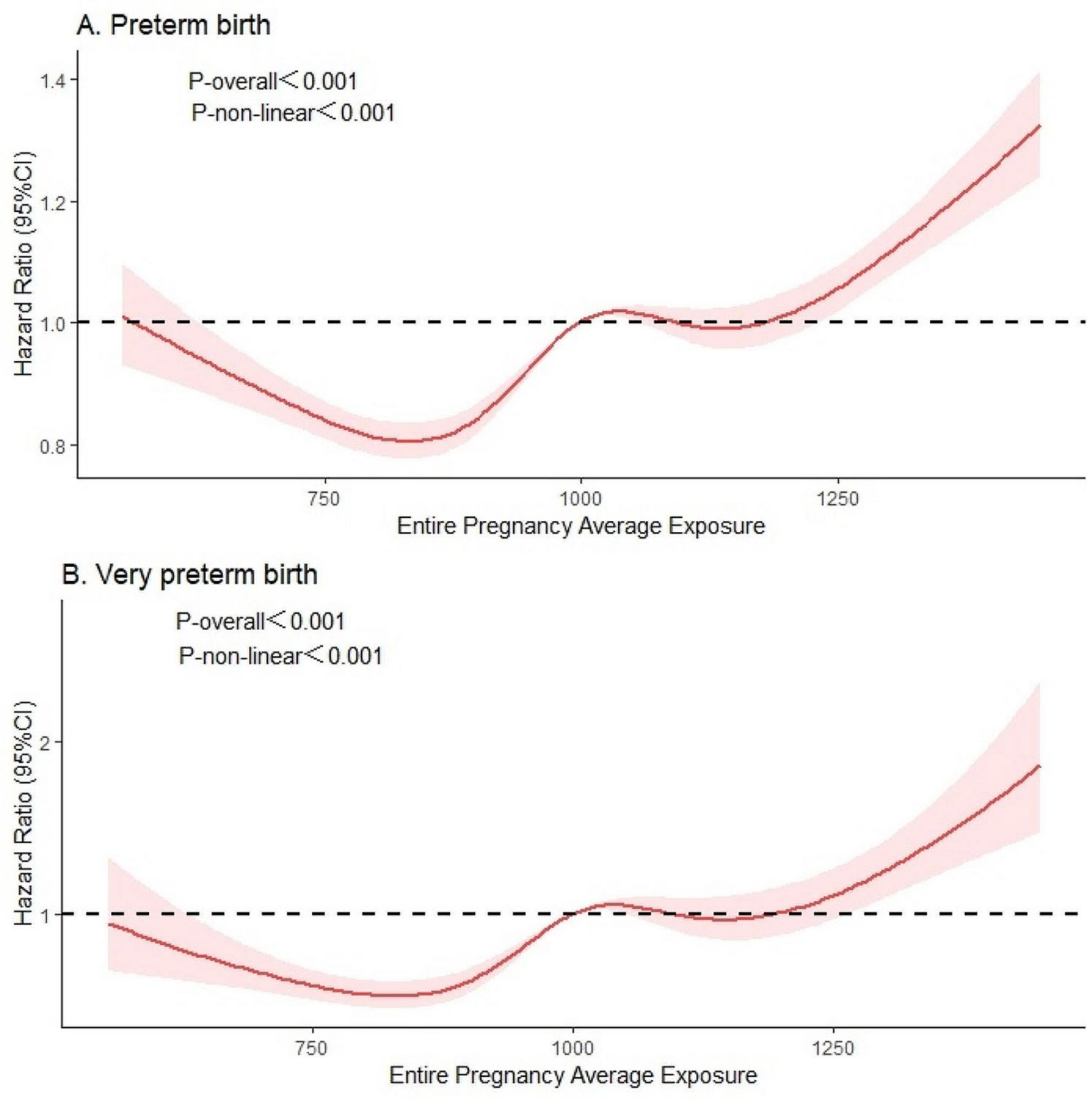
Dose-response associations (smoothing by RCS function with five knots) of preterm and very preterm with ambient CO exposure during the entire pregnancy, with the mean of CO exposure serving as the reference value. Models were adjusted for temperature and relative humidity. Hazard risk was represented by a bold line, and the 95% confidence interval was represented by the shaded area

The results of restricted cubic spline (RCS) models, presented in Fig. 5, further confirmed the non-linear dose-response association between ambient CO exposure and the risk of preterm and very preterm births during the entire pregnancy. Elevated CO concentrations above thresholds were positively associated with an increased risk of preterm and very preterm births. HRs for preterm and very preterm birth at the 95th percentile of CO, compared to the thresholds, were 1.150 (95%CI: 1.105–1.196) and 1.345 (95%CI: 1.169–1.548), respectively. However, HRs for preterm and very preterm birth at the 25th percentile of CO, compared to the thresholds, were 0.928 (95%CI: 0.914–0.942) and 0.806 (95%CI: 0.760–0.855), respectively.

**Table 2:** Hazard risk and 95%CI of preterm birth associated with a 100 µg/m^3^ increment in ambient CO concentrations

Pregnancy period		Trimester 1	Trimester 2	Trimester 3	Entire pregnancy
Model1		1.027(1.022,1.032)	1.033(1.028,1.038)	1.019(1.014,1.024)	1.046(1.040,1.053)
Model2		1.054(1.048,1.060)	1.066(1.060,1.073)	1.007(1.001,1.013)	1.080(1.073,1.088)
Model3	CO and PM_2.5_				
	CO	1.100(1.091,1.110)	1.133(1.123,1.143)	1.049(1.040,1.058)	1.140(1.128,1.152)
	*P*-value interaction	<0.001	<0.001	<0.001	<0.001
	CO and PM_10_				
	CO	1.104(1.093,1,115)	1.162(1.150,1.174)	1.048(1.037,1.059)	1.161(1.146,1.176)
	*P*-value interaction	<0.001	<0.001	<0.001	<0.001
	CO and NO_2_				
	CO	1.099(1.089,1.110)	1.133(1.122,1.145)	1.001(0.991,1.011)	1.129(1.117,1.142)
	*P*-value interaction	<0.001	<0.001	<0.001	<0.001
	CO and SO_2_				
	CO	1.093(1.086,1.101)	1.102(1.094,1.110)	1.080(1.071,1.088)	1.120(1.111,1.130)
	*P*-value interaction	<0.001	<0.001	<0.001	<0.001
	CO and O_3_				
	CO	1.051(1.045,1.057)	1.068(1.061,1.076)	1.029(1.022,1.035)	1.092(1.083,1.101)
	*P*-value interaction	<0.001	0.081	<0.001	<0.001

*Note* Model 1 was unadjusted; Model 2 was adjusted for temperature and relative humidity; Model 3 was adjusted for interact term, temperature and relative humidity

**Table 3:** Hazard risk and 95%CI of very preterm birth associated with a 100 µg/m^3^ increment in ambient CO concentrations

Pregnancy period		Trimester 1	Trimester 2	Trimester 3	Entire pregnancy
Model1		1.080(1.061,1.099)	1.078(1.059,1.097)	1.029(1.010,1.048)	1.130(1.103,1.158)
Model2		1.151(1.126,1.177)	1.147(1.120,1.174)	0.991(0.968,1.015)	1.227(1.193,1.262)
Model3	CO and PM_2.5_				
	CO	1.261(1.221,1.301)	1.312(1.269,1.357)	1.125(1.084,1.167)	1.389(1.335,1.446)
	*P*-value interaction	<0.001	<0.001	<0.001	<0.001
	CO and PM_10_				
	CO	1.279(1.232,1.327)	1.381(1.327,1.437)	1.137(1.087,1.189)	1.456(1.386,1.531)
	*P*-value interaction	<0.001	<0.001	<0.001	<0.001
	CO and NO_2_				
	CO	1.274(1.228,1.322)	1.357(1.301,1.416)	0.961(0.924,0.998)	1.389(1.325,1.456)
	*P*-value interaction	<0.001	<0.001	0.045	<0.001
	CO and SO_2_				
	CO	1.230(1.199,1.262)	1.229(1.195,1.265)	1.215(1.175,1.256)	1.320(1.275,1.367)
	*P*-value interaction	<0.001	<0.001	<0.001	<0.001
	CO and O_3_				
	CO	1.144(1.118,1.170)	1.160(1.131,1.190)	1.024(0.997,1.051)	1.239(1.199,1.281)
	*P*-value interaction	0.009	0.010	<0.001	0.285

*Note* Model 1 was unadjusted; Model 2 was adjusted for temperature and relative humidity; Model 3 was adjusted for interact term, temperature and relative humidity

## Discussion

To our knowledge, this study represents the first extensive population-based investigation addressing both acute and chronic effects of ambient carbon monoxide (CO) on preterm and very preterm birth. Our results indicated that exposure to ambient CO has an acute effect on preterm birth at lag 0–3 and lag 12–21 days, with no significant impact on very preterm birth. Notably, a protective effect on preterm birth was observed at lag 0 day for CO concentrations below the threshold. Concerning long-term effects, increased CO concentrations during each trimester and throughout the entire pregnancy were associated with an elevated risk of both preterm and very preterm birth.

Despite CO being widely recognized as a common cause of poisoning and a routinely monitored air pollutant, our threshold analyses reveal that both acute and chronic exposure to low-level CO was associated with a reduced risk of preterm birth, with long-term low-dose exposure being protective against very preterm birth. This suggests that the effects of CO may vary at different exposure levels. Ritz et al. reported a negative association between entire pregnancy CO exposure and preterm birth (0.59–0.91 ppm carbon monoxide: OR = 0.76, 95%CI: 0.62–0.97) [20]. Furthermore, existing research indicates that the inhalation of CO at a concentration of 125 ppm for 2 h per day over 4 days did not exceed a 4.5% increase in carboxyhemoglobin (CO-Hb) level [21]. The adverse effects associated with CO exposure are typically observed only when the CO-Hb level reaches approximately 20%. Furthermore, it has been found that expectant mothers exposed to ambient CO levels independently exhibit a decreased risk of developing pre-eclampsia. Several studies suggest that the underlying biological mechanism through which low concentrations of CO protective effects against preterm birth [22]. Olgun NS et al. reported that ET-1 is associated with preterm birth [23]. Peltier MR et al. found that the introduction of a low, non-toxic level of 250 ppm CO can prevent the production of 1 L-1β in placental explants stimulated by bacteria, as well as TNFα in fetal membranes. Both of these substances are known to induce ET-1 [24, 25]. Other studies have also demonstrated the impact of CO on reducing the activity of the ET axis in various systems [26, 27], such as mitigating the increase in ET-1 production caused by an infection in the human placenta through the administration of low doses of CO [26].

Moreover, a threshold value of air pollutant effect is usually expected to protect population health by keeping the pollutant below this level. Higher levels of CO exposure throughout pregnancy were found to be associated with an increased likelihood of preterm and very preterm birth. For short-term effect, the log(RR) of preterm birth was 0.160 (95%CI: 0.115–0.205) at the 95th percentile of CO compared to the thresholds. For long-term effect, the hazard risk of preterm birth was 1.150 (95%CI: 1.105–1.196) at the 95th percentile of CO compared to the thresholds, while the risk of very preterm birth was 1.345 (95%CI: 1.169–1.548). This aligns with a study in Brisbane that associated increased CO concentrations above 162.5 ppb with an increased risk of preterm birth [28]. While our study reveals a protective effect of low-dose CO exposure on preterm and very preterm birth, it is essential to note that short-term exposure to CO increases the risk of preterm birth but not very preterm birth. Additionally, long-term exposure to CO further elevates the risk of preterm and very preterm birth when considering the overall CO exposure dose.

Our findings align with some previous research in confirming the acute association between CO exposure and the risk of preterm birth. Discrepancies between our study and others may arise from variations in exposure time-frame and effect estimates. For instance, a time-series study in Ningbo found that the largest excess risks (ERs) were at lag 4 for CO (ERs = 3.36, 95%CI: 0.5–6.3) [29]. In our study, preterm birth was associated with CO when exposure lagged 0–3 days and 12–21 days, while very preterm birth did not exhibit a similar pattern. Regarding the long-term impact of CO, our study establishes a correlation between CO exposure and each trimester of preterm and very preterm birth. In addition, we found the hazard risk of both preterm and very preterm increased from low-to-mean concentrations of CO, followed by a continued increase from mean-to-high concentrations (Fig. 5). However, existing literature on the relationship between CO, preterm, and very preterm birth offers mixed results. Gong et al. demonstrated that exclusive exposure to CO during the third trimester significantly increases the risk of preterm birth [30]. A prospective birth cohort study found a 15% (OR = 1.15, 95%CI: 1.11, 1.19) increase in the risk of preterm birth with 100 µg/m^3^ increase in CO concentration during the entire pregnancy, with the strongest effect in the second trimester for CO [17]. The results of a study in the U.S. State of Georgia suggest that there is an association between carbon monoxide (CO) exposure and preterm birth. The odds ratios for inter-quartile range increases in CO during the first, second, and third trimesters as well as throughout the entire pregnancy were found to be 1.005 (95%CI: 1.001–1.009), 1.007 (95%CI: 1.002–1.011), and 1.011 (95%CI: 1.006–1.017), respectively [31]. However, among about 3509 women delivering between 1996 and 2006, Rudra et al. found no evidence that CO influences the risk of preterm delivery in western Washington [32]. Similarly, Ju et al. reported no significant association between CO exposure and increased risk of very preterm birth (RR = 0.930, 95%CI: 0.847–1.022) [33].

Our findings further suggest that air pollutants (PM_2.5_, PM_10_, NO_2_, SO_2_, O_3_) may partially increase the effect of CO exposure on the risk of both preterm and very preterm birth. The multiplicative interaction of air pollutants and CO contributes to the risk of preterm and very preterm birth. This may be attributed to air pollution, particularly PM_2.5_ and PM_10_, inducing oxidative stress and inflammation in the body, thereby augmenting the risk of CO exposure during pregnancy. The effects of CO on preterm birth appeared to be stronger for older women in the first trimester. To further evaluate the impact of CO exposure throughout pregnancy, subgroup analysis by maternal age should be conducted.

Despite offering new insights into the short-term and long-term effects of CO on preterm and very preterm birth, our study has several limitations. Firstly, due to data unavailability, we are unable to account for more detailed demographic information (e.g., family income, occupation and education level) [16, 34] and other behavioral risk factors related to preterm and very preterm birth outcomes (e.g., parental smoking, diet habits) [35]. We didn’t use multiple exposure methods to analyze the effects of CO and other air pollutants exposure on preterm birth. However, the population-based nature of our study minimizes participant selection bias, and the large population and extended study period enhance the accuracy of the estimated impact. Secondly, the presence of indoor air pollution resulting from cooking practices and traditional heating methods in rural regions could potentially emerge as a noteworthy environmental element [36]. Moreover, CO concentrations were measured based on monitor station rather than individual exposure, and we were unable to accurately calculate the exposure of pregnant women to activity trajectories, work, etc. Nevertheless, we employed the inverse distance weighting technique to estimate the various long-term exposure periods for each pregnant woman based on registered addresses. This encompassed different time intervals and the cumulative average concentration of CO throughout the follow-up duration.

## Conclusion

In summary, our findings suggest that short-term exposure to low levels of CO may have protective effects against preterm birth, while long-term exposure to low concentrations of CO may reduce the risk of both preterm and very preterm birth. Moreover, our study provides evidence indicating that very preterm birth is more susceptible to the influence of long-term exposure to CO during pregnancy, with acute CO exposure exhibiting a greater impact on preterm birth. Despite the comprehensive nature of our large population-based study, the assertion regarding the protective effect of low-dose CO exposure on preterm and very preterm birth necessitates careful consideration. Further investigations into the underlying biological mechanisms are imperative to substantiate this conclusion. It is imperative for pregnant women to minimize exposure to ambient air pollutants.

### Electronic supplementary material

Below is the link to the electronic supplementary material.

Supplementary Material 1

## Data Availability

The data sets generated and analysed during the current study are not publicly available due property rights protection but are available from the correspinding author on resonable request.
