# Supplementary material for: Association between risk of preterm birth and long-term and short-term exposure to ambient carbon monoxide during pregnancy in chongqing, China: a study from 2016-2020
Source: BMC Public Health. 2024 May 27;24:1411. doi: 10.1186/s12889-024-18913-z (PMC11129390; doi:10.1186/s12889-024-18913-z)
Supplement: Supplementary file 1 — Supplementary Material 1 [file 12889_2024_18913_MOESM1_ESM.docx]

**Supplementary Material**

Table S1. The overall distribution of preterm birth, very preterm birth, air pollution and meteorological factors.

| Variable | Mean±SD | Min | Max |  | Percentile | | |
| --- | --- | --- | --- | --- | --- | --- | --- |
|  |  |  |  |  | 25th | 50th | 75th |
| Total birth | 282.465±104.374 | 51.000 | 1052.000 |  | 212.000 | 264.000 | 324.000 |
| Preterm birth | 16.923±7.182 | 0.000 | 48.000 |  | 12.000 | 16.000 | 22.000 |
| Very preterm birth | 1.162±1.294 | 0.000 | 8.000 |  | 0.000 | 1.000 | 2.000 |
| Air pollution |  |  |  |  |  |  |  |
| PM_2.5_ (μg/m^3^) | 40.735±2.310 | 7.471 | 165.941 |  | 24.953 | 35.149 | 49.632 |
| PM_10_ (μg/m^3^) | 64.003±31.065 | 13.059 | 225.177 |  | 41.941 | 57.485 | 76.941 |
| SO_2_ (μg/m^3^) | 9.376±3.700 | 3.765 | 31.471 |  | 6.765 | 8.412 | 11.078 |
| NO_2_ (μg/m^3^) | 40.249±11.263 | 12.235 | 81.824 |  | 32.059 | 38.412 | 47.294 |
| O_3_ (μg/m^3^) | 42.368±26.264 | 4.412 | 14.265 |  | 21.441 | 37.176 | 57.882 |
| CO (μg/m^3^) | 886.521±205.966 | 466.471 | 2975.882 |  | 750.529 | 849.412 | 981.824 |
| Meteorological factors | |  |  |  |  |  |  |
| Temperature (℃) | 19.972±7.972 | 1.200 | 36.500 |  | 12.600 | 20.300 | 26.500 |
| Relative humidity (%) | 75.105±11.821 | 37.000 | 97.000 |  | 67.000 | 76.000 | 84.000 |


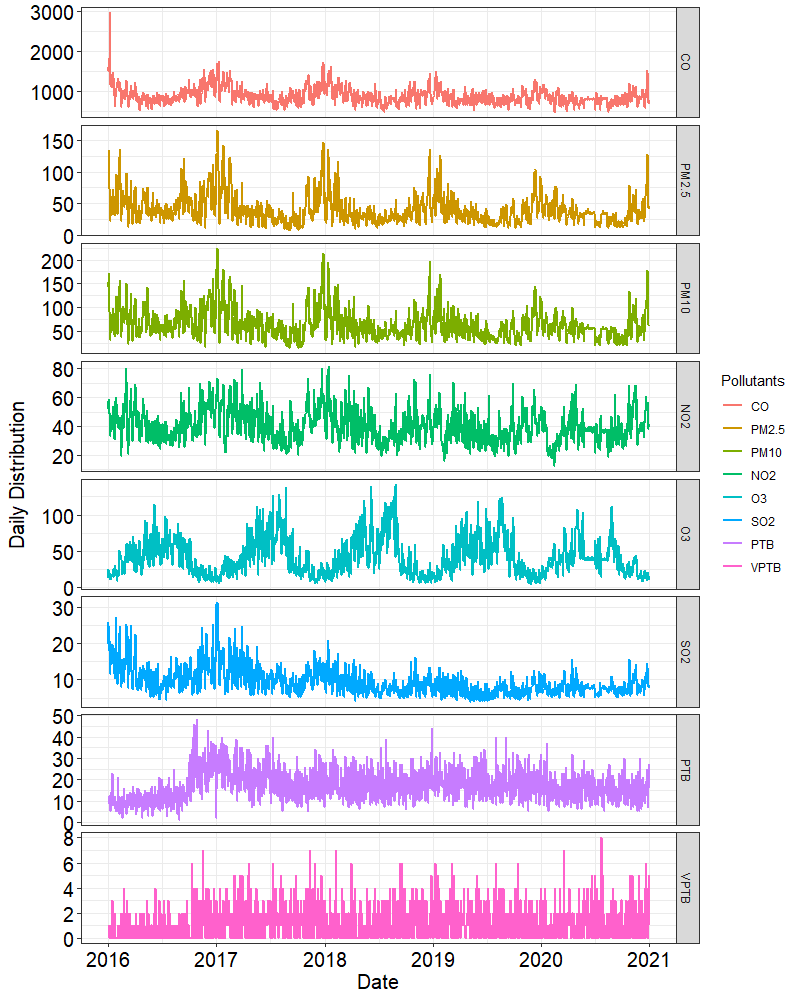


Supplementary figure1. The distribution of air pollutant concentrations and daily preterm birth in Chongqing, China from 2016 to 2020.

Note: CO (μg/m^3^) = carbon monoxide, PM_2.5_ (μg/m^3^) = particulate matter particulate matter≤2.5μm in aerodynamic, PM_10_ (μg/m^3^) = particulate matter particulate matter ≤10 μm in aerodynamic, NO_2_ (μg/m^3^) = nitrogen dioxide, O_3_ (μg/m^3^) = ozone, SO_2_ (μg/m^3^) = sulphur dioxide.
